# Valorisation of Broccoli By-Products: Technological, Sensory and Flavour Properties of Durum Pasta Fortified with Broccoli Leaf Powder

**DOI:** 10.3390/molecules27154672

**Published:** 2022-07-22

**Authors:** Natalia Drabińska, Mariana Nogueira, Beata Szmatowicz

**Affiliations:** 1 Department of Chemistry and Biodynamics of Food, Institute of Animal Reproduction and Food Research of Polish Academy of Sciences, Tuwima 10, 10-748 Olsztyn, Poland; s-masanogueira@ucp.pt; 2 Faculty of Biotechnology, Catholic University of Portugal, 4169-005 Porto, Portugal; 3 Sensory Laboratory, Institute of Animal Reproduction and Food Research of Polish Academy of Sciences, 10-748 Olsztyn, Poland; b.szmatowicz@pan.olsztyn.pl

**Keywords:** broccoli leaves, by-products, valorisation, pasta, sensory analysis, volatile compounds

## Abstract

The aim of this study was to evaluate the effect of broccoli leaf powder (BLP) incorporation on the technological properties, sensory quality and volatile organic compounds (VOCs) of durum wheat pasta. Incorporation of BLP increased cooking loss; however, all pasta samples were found to be in the acceptable range of 8 g/100 g. The addition of BLP decreased optimal cooking time and water absorption but increased the swelling index. Firmness and total shearing force decreased with increased BLP content. The obtained pasta was greener than the control, with a higher content of minerals, and an increasing tendency with respect to protein was observed. The VOC profile of enriched pasta was richer and contained compounds typical of broccoli (e.g., dimethyl sulphide), affecting its aroma. The sensory evaluation results indicate that the addition of BLP did not affect the overall acceptance of pasta. Up to 5% BLP content afforded an interesting, more nutritious pasta without compromising its technological and sensory quality.

## 1. Introduction

The Western diet is rich in highly processed products, which can contribute to the development of noncommunicable diseases. Consumers are becoming increasingly aware of the importance of diet with respect to the maintenance of a healthy lifestyle and the development of noncommunicable diseases. Nevertheless, the consumption of vegetables does not meet the dietary guidelines very often [1,2]. Therefore, solutions that promote an increase in vegetable intake are in demand. Wheat-based products, due to their high daily consumption, have been repeatedly proposed for functional food development. Among cereal products, pasta seems to be a good vehicle for the incorporation of nutritional ingredients, as it is a basic product commonly eaten worldwide by members of all social groups and is also eagerly selected by children. Moreover, pasta has a low price and relatively long shelf life and can maintain acceptable physical and sensory properties when new ingredients are added [3,4,5,6]. In the recent years, many studies have focused on the incorporation of various ingredients into pasta, including vegetables, legumes, pseudo-cereals and animal proteins [7,8,9,10]. In general, the obtained results have shown an improvement in nutritional properties and antioxidant activity of the final, functional products. However, the replacement of semolina is still a challenge for the food industry, as modification in the formulation can affect pasta quality in terms of texture, colour, technological quality and sensory properties [11].

The fruit and vegetable processing industry is one of the largest producers of by-products [12]. Many by-products from fruit and vegetable processing can be used as sources of nutrients and functional ingredients, increasing the added value of products without increasing production costs [13,14,15,16,17,18]. Each year, a significant portion of cruciferous crops are not harvested or utilized [19]. With respect to broccoli, only 10–15% of the total aerial biomass of the plant is consumed. The possible consumption of stems and leaves could facilitate the management of vegetable processing waste and increase the productivity and sustainability of the global broccoli crop [19]. Although broccoli leaves are rarely acceptable for consumption, studies have reported that similarly to the edible parts, leaves are rich sources of bioactive compounds (glucosinolates and polyphenols) and other essential nutrients, such as vitamins E and K [19,20,21]. Broccoli by-products have been used traditionally as an animal feedstuff and as antimicrobial agents for foodborne or soilborne bacteria [19]. However, broccoli leaves have gained scientific attention in recent years, mainly due to their bioactive compounds [20,22,23,24]. The successful incorporation of broccoli leaves has been reported in recently developed gluten-free mini sponge cakes, gluten-free bread and beverages [20,25,26]. However, to the best of our knowledge, broccoli leaves have never been used to enrich pasta products.

Therefore, we attempted to incorporate broccoli leaf powder (BLP) into another type of product obtained from different ingredients and processed differently, namely durum pasta. Compared to bakery goods, lower temperatures are applied for pasta production, which can potentially preserve the bioactive and aroma compounds in fortified pasta products. The aim of this study was to evaluate the effect of BLP on the technological properties, sensory quality and volatile organic compounds (VOCs) of durum wheat pasta.

## 2. Results and Discussion

In this study, three formulations were compared, including control (C; not fortified with BLP; B2.5, fortified with 2.5% BLP; and B5, fortified with 5% BLP (Table 1).

### 2.1. Cooking Properties

Cooking quality is an important attribute affecting consumer acceptance. The cooking properties of the investigated pasta products are shown in Table 2. In terms of optimal cooking time (OCT) and cooking losses, C and B5 pasta were very similar. Pasta B2.5 had significantly shorter OCT and higher values of cooking loss. A decreasing trend was observed in terms of water absorption; however, the differences were not statistically significant. Control pasta had a significantly lower swelling index (SI) compared to BLP-fortified pasta.

In this study, the OCT varied between 3.0 and 3.6 min, which is a relatively small range. Incorporation of BLP resulted in a reduction in OCT, which can be explained by the high water absorption index of freeze-dried BLP [27]. Significant changes were observed only in pasta B2.5, probably due to higher moisture content and, consequently, faster rehydration. Similar results were observed by Michalak-Majewska et al. [11], who reported that pasta fortified with onion skin powder had a shorter OCT compared to control pasta. Silva et al. [3] reported that the OCT of fresh pasta enriched with concentrations of broccoli up to 30% varied between 2.5 and 4.5 min, which is a wider range than that observed in this study. Water absorption also decreased with the addition of BLP, although the differences were not statistically significant. This could be attributed to shorter cooking times, which can reduce the water absorption of pasta [11]. 

Another parameter defining cooking properties analysed in this study is cooking loss. Cooking loss represents the amount of solid material lost in the cooking water and is one of the most important parameters determining the quality of pasta [10]. In this study, the highest cooking loss was observed for B2.5 pasta, whereas control and B5 had significantly lower cooking losses. Importantly, none of the experimental pasta exceeded the technologically acceptable limit of 8% [10], indicating that incorporation of BLP into pasta does not decrease the quality of pasta. Michalak-Majewska et al. [11] reported that cooking losses remained below 8% for pasta fortified with 2.5%, 5% and 7.5% onion by-product. These findings suggest that adding vegetable by-products in these concentrations might be a good option to enrich pasta products with vegetables without compromising pasta quality. In contrast, the incorporation of fruit by-products, namely 5% mango peel powder, resulted in cooking losses of more than 8%, which could result from the high sugar content in mango peel powder itself [28].

SI of pasta is a good indicator of the integrity of the protein matrix, which restricts water penetration and depends on competition between the starch and protein for water absorption [5,6]. In this study, pasta C had the lowest SI among all analysed pasta. The addition of BLP did not inhibit the swelling of the starch granules, but it seems that BLP contributed to the formation of a protein network, which increased the supply of water for swelling and gelatinisation of starch granules. A previous study showed that the incorporation of protein into the pasta formulation resulted in a reduction in SI [6]. On the other hand, some studies reported a significant increase in the SI with increased concentrations of dietary fibre [29]. Although the content of dietary fibre was not evaluated in this work, based on the results of a previous study reporting high content of dietary fibre in broccoli leaves [27], it can be assumed that it could also affect the SI of experimental pasta.

### 2.2. Texture

Textural parameters of experimental pasta are shown in Table 2. Fortification of pasta with BLP resulted in a decrease in firmness and total shearing force (TSF) with increased BLP percentage. No significant differences were observed between B2.5 and B5 samples; however, a decreasing tendency was observed.

The texture of pasta is an important parameter influencing consumer acceptance. Pasta firmness is defined as the peak force attained during the first compression and is closely related to the strength and integrity of the protein matrix developed during the cooking process, whereas TSF is defined as the force to cut/shear [10,30]. The present study showed that the incorporation of BLP into pasta decreased both textural parameters. Firmness can be reduced as a result of a higher SI [6], which was observed in BLP-fortified pasta. Similar findings were reported by Gull et al. [31], who noted a decrease in the pasta firmness after the incorporation of carrot pomace. Based on the results reported by Silva et al. [3], who showed that the biggest changes in texture were observed after the addition of 20% to 30% of broccoli floret, it can be assumed that the concentration of BLP used in this study does not compromise the texture quality of pasta. 

### 2.3. Proximal Composition

The proximate chemical composition of BLP and experimental pasta products is shown in Table 2. BLP was found to be a rich source of protein and minerals. The fat content of BLP was relatively low, accounting for approximately 4%. The obtained results are similar to those obtained by other authors [20,27].

The incorporation of BLP into pasta resulted in a gradual increase in mineral content, together with an increasing percentage of BLP. The protein content of fortified pasta also increased; however, the differences were not statistically valid. Pasta B2.5 had the highest content of fat, followed by B5 and C. The moisture of the experimental pasta was significantly higher in B2.5, whereas that of B5 and C were similar. As a consequence of changes in other fractions, the estimated carbohydrate content was the lowest in B2.5. 

In this study, a significant increase in ash content was observed as a result of a higher mineral content delivered by BLP, which is in agreement with a previous study showing that broccoli leaves had a higher content of ash compared to florets [27]. The incorporation of broccoli has been previously reported to increase protein, fat and mineral contents in bread [25,32]. Although BLP was found to be a good source of protein, in this study, only an insignificant increase was observed, which can be explained by the fact that semolina used for pasta production is itself a good source of protein; because the BLP contribution to the pasta formulation was small, it was not possible to notice its effect. On the other hand, the content of ash in semolina is low; thus, the addition of mineral-rich BLP caused a more pronounced effect. Notably, the addition of BLP to the pasta also resulted in a significant increase in fat content. In general, broccoli is considered a low-fat product. However, it has been reported that broccoli leaves are a rich source of polyunsaturated fatty acids, mainly α-linolenic, linoleic and palmitic acids [27]. The profile of fatty acids was not analysed in this study, mainly due to the low fat content in broccoli; however considering the increase in fat content in enriched pasta, it is worth considering fatty acid content in future studies.

### 2.4. Pasta Colour

**Figure 1:**
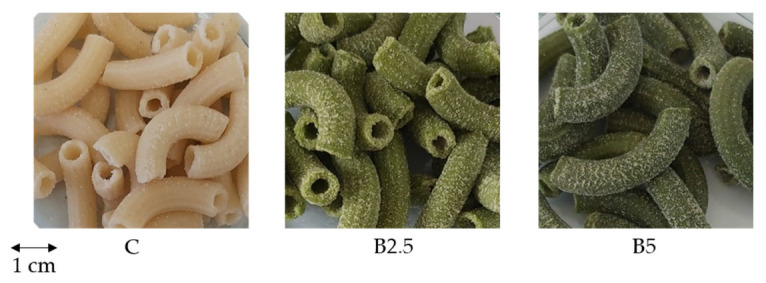
Experimental pasta fortified with BLP. C: control pasta; B2.5: pasta fortified with 2.5% broccoli leaf powder; B5: pasta fortified with 5% broccoli leaf powder.

The colour characteristics of BLP and experimental pasta are presented in Table 2. As expected, BLP was the greenest among the analysed samples. Fortification of pasta with BLP significantly decreased lightness (L* value) and increased yellowness (b* value) and greenness (a* value). No statistically significant difference in terms of greenness was noted between B2.5 and B5 pasta, although B5 seemed to be greener (Figure 1). The whiteness index (WI) significantly decreased with increased addition of BLP, whereas the browning index (BI) significantly increased.

Pasta colour is an important factors influencing consumer acceptance and is associated with product freshness and flavour expectations [33]. In recent years, pasta products with unconventional colours have gained attention, and the use of natural colourants is increasingly accepted by consumers [33]. In this study, the lightness (L* value) of pasta samples decreased with increased BLP content, which is in agreement with other studies on the fortification of semolina-based products with plant-based ingredients [4,33]. The addition of BLP to pasta also resulted in a decrease in a* values. This was expected, as the −a* parameter represents the greenness of the analysed sample, and BLP is characterized by high green intensity (a* = −9.33 ± 0.03). As a consequence, in L* and a* parameters, the WI significantly decreased, and BI increased with the addition of BLP. Although the green appearance of pasta could cause some concern for consumers not used to purchasing this kind of product, the current tendency towards “healthier” foods may represent an opportunity to introduce green pasta to the market [4]. 

### 2.5. Sensory Analysis

**Figure 2:**
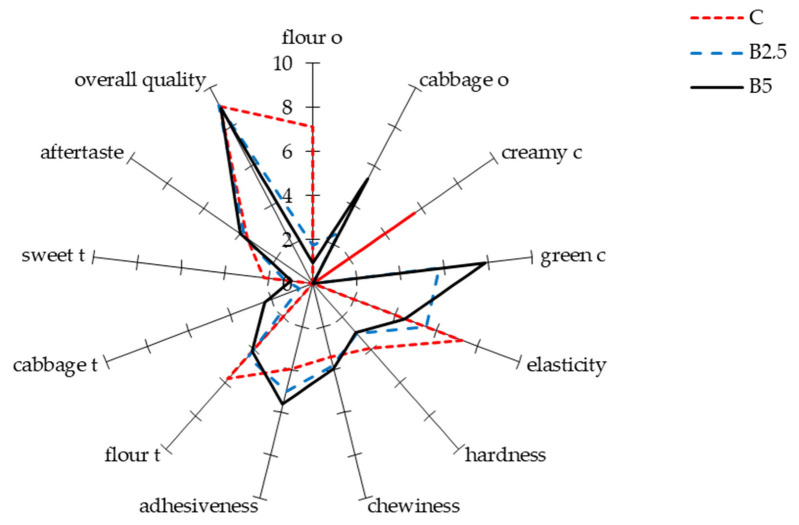
QDA sensory analysis and PCA biplot of experimental pasta fortified with BLP. C: control pasta; B2.5: pasta fortified with 2.5% broccoli leaf powder; B5: pasta fortified with 5% broccoli leaf powder; o: odour; c: colour; t: taste.

The results of the sensory evaluation of experimental pasta types using the quantitative descriptive analysis (QDA) method are presented in Figure 2. Panellists did not notice any difference in the hardness or chewiness of pasta. However, the degree of adhesiveness significantly increased, and elasticity decreased with increased BLP content. Cabbage-like odour and taste increased with increased BLP content; however, it did not negatively affect panellists’ perception of the pasta. The incorporation of BLP did not affect the overall acceptance of experimental pasta products, which were rated very high (9 on a 10-point scale). Based on the obtained results, it can be concluded that pasta fortified with up to 5% BLP remains acceptable by consumers in terms of taste, odour, appearance and texture. According to a study by Silva et al. [3], the incorporation up to 20% broccoli florets in pasta did not deteriorate its acceptability. The authors reported that the intensity of the vegetable flavour increased with increased broccoli content, which was also observed in this study. On the contrary, incorporation of BLP (even 2.5%) as a starch replacement in gluten-free sponge cakes reduced the overall acceptance [20]. This could be due to the fact that the sweet pastry products may not associate well with vegetables.

The results of sensory analysis in terms of texture corresponded to the results of instrumental texture analysis. The highest firmness was noted for pasta C, which resulted in the highest hardness as evaluated by the sensory panel. The incorporation of BLP resulted in a decrease in firmness/hardness of experimental pasta, although the values were not significant. It can be assumed that fortification of pasta with higher BLP content could cause more substantial changes; for example, Silva et al. [3] reported a significant decrease in hardness after incorporation of up to 30% broccoli florets.

Chewiness and adhesiveness can be associated with cooking properties, specifically with OCT and cooking loss, respectively [5]. Although OCT differed between the samples, the changes were not considerable enough to affect the chewiness. On the contrary, the incorporation of chickpea flour as a substitute for semolina resulted in a decrease in chewiness of pasta [7]. The adhesiveness of the experimental pasta increased with increased BLP content, which is in agreement the increased cooking loss observed in pasta B2.5. However, increased BLP content cannot explain changes in adhesiveness reported in pasta B5, which exhibited a similar cooking loss to pasta C. This can be explained by the higher SI of pasta B5 and the increment of starch gelatinisation, as well as the consequent adhesiveness [29]. The obtained results are in agreement with a those of a previous study showing that the incorporation of up to 7.5% onion by-products into pasta decreased the adhesiveness [11]. On the contrary, Silva et al. [3] concluded that incorporation of up to 30% broccoli did not affect the stickiness of pasta.

The decrease in the elasticity of pasta with increasing BLP content could be associated with lower TSF values. Previous studies showed that dietary fibre supplementation decreased pasta elasticity [6]. For instance, the addition of brewers’ spent grains (the main by-product of the brewing industry) to pasta significantly decreased the elasticity of the dough [34]. Although the content of fibre was not evaluated in this study, it is likely that BLP has a higher fibre content than semolina, which could explain the decrease in pasta elasticity.

### 2.6. Volatile Organic Compounds

Aroma has a considerable influence on consumer acceptability [35]. VOCs contribute to the flavour and aroma of food products and are key factors determining perceived quality. Therefore, the profiling of secondary metabolites, such as VOCs, by analytical techniques is widely used in food quality assessment [36]. In this study, VOC composition was investigated both in BLP and in cooked pasta using solid-phase microextraction (SPME) with gas chromatography-mass spectrometry (GC-MS). 

In this study, only VOCs with identity of the highest threshold and that could be associated with odour descriptors [37] were included in the analysis (Table 3). In total, 66 aroma-active compounds were identified, including 21 aldehydes, 15 ketones, 14 alcohols, 2 sulphur compounds, 6 terpenes, 2 alkanes, 4 furan derivatives and 2 other compounds. BLP was characterized by a rich profile of VOCs (52 compounds), including 16 aldehydes, 14 ketones, 8 alcohols, 2 sulphur compounds, 5 terpenes, 2 alkanes and 4-(2-propenyl)-phenols. The most abundant VOCs in BLP were (*E,E*)-3,5-octadien-2-one, 1-penten-3-ol, 6-methyl-5-hepten-2-one and (*Z*)-2-penten-1-ol. Compared to the C pasta, the total abundance of VOCs in BLP was 8.5 times higher. Only 29 VOCs were detected in C pasta, with aldehydes being the most numerous group (12 compounds). The most abundant VOCs in C pasta were hexanal, 1-hexanol, limonene and 2-pentyl-furan. In pasta fortified with BLP, the presence of 59 compounds was detected, including 30 VOCs derived from BLP, all originating exclusively from C (14 VOCs), whereas 15 compounds were present in both C and BLP. Consequently, the odour descriptions of cabbage, pungent, sulphur, rancid, malty and spicy could be assigned to BLP and fortified pasta but not to C pasta. The most abundant VOCs in B2.5 were hexanal, 1-hexanol, 1-octen-3-ol and limonene, whereas in B5, the same compounds were the most abundant but in a different order (hexanal > limonene > 1-hexanol > 1-octen-3-ol). Statistical comparison showed that the abundance of 19 compounds, including 8 aldehydes, 5 ketones, 4 terpenes and 2 furan derivatives, increased with the increased addition of BLP, which resulted in a significant increase in the total peak area in pasta B5 (Table 3). The odour descriptions of these compounds were mainly sulphurous, green, rancid, fruity and floral.

Although the VOCs in Brassica vegetables have been widely studied [38,39,40,41], in this study, the profile of broccoli leaves was analysed for the first time. Previous studies showed that the analysis of fresh vegetables resulted in a high abundance of sulphur compounds, including isothiocyanates derived from glucosinolates, which are responsible for a cabbage-like aroma [38,39]. On the contrary, in this study, no isothiocyanates were detected. Isothiocyanates are products of the enzymatic hydrolysis of glucosinolates; therefore, a lack of these compounds can be explained by the inactivation of myrosinase during the blanching step in BLP preparation. Moreover, isothiocyanates are high volatility and could possibly be evaporated during freeze drying. It should also be noted that analysis of isothiocyanates by SPME is challenging and possible only in high abundances.

Various odour descriptors could be associated with compounds detected in BLP, with fruity and green being the most frequent. An important compound responsible for sulphur aroma is dimethyl sulphide. Although its abundance was low in BLP and did not differ between pastas B2.5 and 5, this compound was previously reported to be one of the key odorants in Brassica vegetables [41]. Importantly, the odour threshold of dimethyl sulphide is very low and was estimated as 0.0008–0.03 and 0.0003–0.16 mg kg^−1^ in water and air, respectively [42]. Low odour thresholds indicate odours are detectable at extremely low concentrations—very often below the limit of detection [41]. Therefore, despite its low abundance, it can be assumed that this compound was responsible for the cabbage-like aroma detected by the sensory panel. On contrast, the odour thresholds in water for the most abundant compounds in BLP were estimated as 0.15, 0.40, 0.05–8.2 and 0.72 for (*E,E*)-3,5-octadien-2-one, 1-penten-3-ol, 6-methyl-5-hepten-2-one and (*Z*)-2-penten-1-ol, respectively.

Semolina and pasta are usually not considered for their aromatic properties. Nevertheless, a total of 29 VOCs were identified in cooked C pasta, and the most frequently associated odours were citrus and fatty. Aldehydes and alcohols were the major chemical groups present, which is in agreement with results reported by Beleggia et al. [35], who investigated the VOCs of cooked pasta. In that research, 29 VOCs were identified, but only 12 were common to those identified in the present study, which highlights the existing differences between semolina and olive oil used to produce pasta. However, in both studies, hexanal was the most abundant aldehyde in cooked pasta samples. The presence of hexanal, which has been associated with oxidation of unsaturated fatty acids [35], can result from the olive oil added to pasta formulations. Oxidation of fatty acids is also the source of other aldehydes, ketones and alcohols detected in this study, which suggests that these compounds were formed during thermal processing of olive oil and pasta [43]. An interesting phenomenon was observed with respect to hexanal when all the pasta formulations were compared. Although BLP was found to be a rich source of hexanal itself, the level of this compound decreased with increased BLP percentage. This can be explained by the presence of other compounds in B2.5 and B5 formulations, which contributed to a richer headspace. It should be noted that SPME is a competitive method with an observed problem of the replacement of polar compounds with non-polar VOCs, which are highly abundant in food matrices with saturation effects [44]. Therefore, the observed difference in the level of hexanal in this study does not necessarily mean that its concentration decreased but that it could be extracted with lower efficiency due to the presence of other compounds.

2-pentylfuran exhibits a medium-strength fruity, green, earthy, beany, vegetable-like odours [45], and it has been reported to be frequently present in samples of cooked pasta [35]. This compound, as well as hexanal, nonanal and benzaldehyde, has a low odour threshold and contributes to not intensive pasta flavour [43]. Notably, limonene was detected in higher concentrations in cooked pasta than in BLP. Because terpenes are known to have distinctive aromatic properties [35], limonene could also contribute to the flavour of pasta. (*E*)-2-nonenal and (*E,E*)-2,4-decadienal, both with a fatty odour, have been reported as the most potent odours in bread crumb [36]. In the present study, these compounds were detected in all types of cooked pasta but not in BLP, which may suggest that these aldehydes are volatiles associated with cereal products. Gaggiotti et al. [45] concluded that pasta of high quality should contain higher content of hexanal, nonanal and 2-nonenal compared to 2-hexenal and pentenal, as the latter are typical of low-quality pasta. This suggests that the pasta obtained in the present study was of high quality.

In pasta fortified with BLP, compounds described as fruity and green were most frequently detected, confirming the contribution of BLP to the overall aroma. The profile of VOCs was strongly affected by the incorporation of BLP, as 30 VOCs derived exclusively from BLP were detected in fortified pasta. These compounds contributed to the appearance of odours, such as cabbage-like, sulphur, pungent, rancid, malty and spicy. Moreover, as shown in Table 3, the abundance of many compounds increased proportionally to the percentage of BLP. Notably, some of compounds were detected at higher concentrations in B2.5 than in B5, which can be partly explained by the difficulties in analysis of food matrices containing high concentrations of non-polar VOCs, which can result in a displacement effect [44].

**Figure 3:**
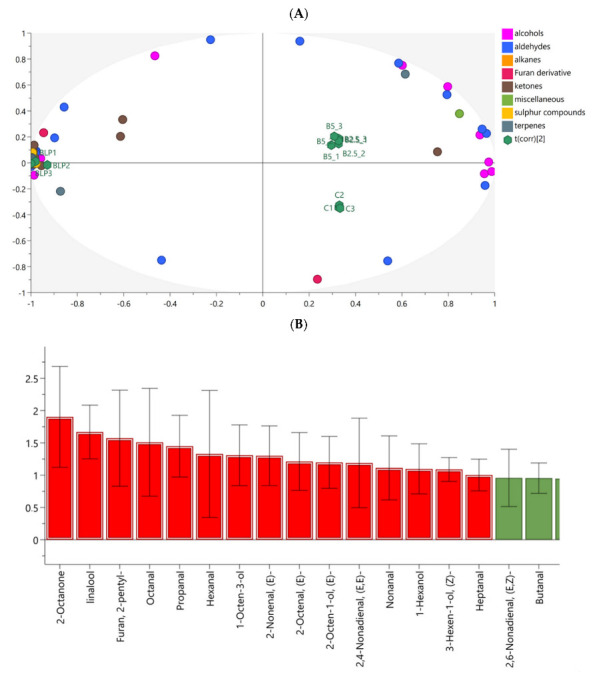
Orthogonal partial least-squares discriminant analysis (OPLS-DA) of experimental pasta and BLP (*R*^2^*X* = 0.966; *R*^2^*Y* = 0.960; *Q*^2^ = 0.810). (**A**) biplot presenting the association between VOCs and samples; (**B**) variable importance in projection (VIP) score. A VIP value > 1.00 indicates the contribution of VOCs in separating the groups (blue bars).

Multivariate analysis confirmed that the most VOCs were associated with BLP (Figure 3A). The following 15 compounds contributed most to the separation between groups: 2-octanone, linalool, 2-pentylfuran, octanal, propanal, hexanal, 1-octen-3-ol, (*E*)-2-nonenal, (*E*)-2-octenal, (*E*)-2-octen-1-ol, (*E,E*)-2,4-nonadienal, nonanal, 1-hexanol and (*Z*)-3-hexen-1-ol (Figure 3B). Compounds associated with BLP and experimental pasta are presented in Figure S1.

### 2.7. Association between Sensory Analysis and Volatile Organic Compounds

**Figure 4:**
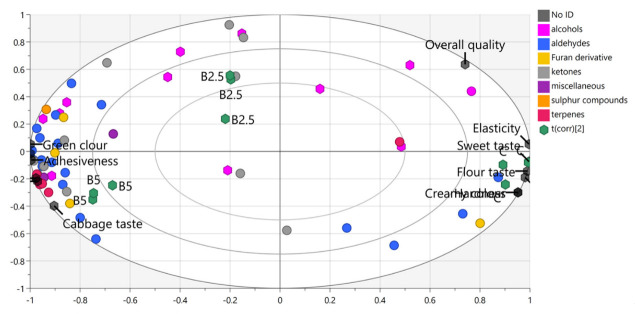
OPLS-DA score plot (*R*^2^*X* = 0.866; *R*^2^*Y* = 0.991; *Q*^2^ = 0.963) obtained for aroma-active VOCs and sensory attributes in experimental pasta. C: control pasta; B2.5: pasta fortified with 2.5% broccoli leaf powder; B5: pasta fortified with 5% broccoli leaf powder.

Orthogonal partial least-squares discriminant analysis (OPLS-DA) was performed to evaluate the overall association between the aroma-active VOCs and sensory descriptors in experimental pasta (Figure 4). Pasta C was located on the right side of the plot and was associated with sensory descriptors (elasticity, hardness, creamy colour, flour odour, flour and sweet taste) and three VOCs (hexenal, 2-pentylfuran and (*E,E*)-2,4-nonadienal). Pasta B2.5 was located in the upper-left quadrant and correlated only with alcohols and ketones (2-octanone, (*Z*)-2-penten-1-ol, acetone, 1-penten-3-ol, 1-nonanol and 4-methyl-2-hexanone). Most VOCs, as well as several sensory descriptors (green colour, chewiness, adhesiveness, aftertaste, cabbage odour and cabbage taste), were associated with pasta B5, which was located in the lower-left quadrant. 

Cabbage flavour detected in pasta B5 could be attributed to the presence of dimethyl sulphide, which was reported to have a high flavour dilution (FD), suggesting that even a small concentration of this compounds contributes to its overall aroma. Because panellists detected a difference in the intensity of cabbage-like aroma between pasta B2.5 and B5, it can be assumed that other sulphur compounds, which usually have relatively low odour thresholds, could be present below the limit of detection [41]. The absence of high-molecular-weight sulphides, such as dimethyl, tri- and tetrasulphides, may be related to the fact that the SPME technique, as an absorption method, is more suitable for the extraction of low-molecular-weight compounds.

## 3. Materials and Methods

### 3.1. Broccoli Leaf Powder

The preparation of BLP was previously described by Drabińska et al. [20]. Briefly, the leaves of broccoli (*Brassica oleracea* L. var. *italica* cv. Sebastian) without any sign of mechanical damage were selected and washed in tap water to remove soil residues. Leaves were blanched in boiling water for 1 min to inactivate enzymes. The petioles and the main midribs were removed, and the material was freeze-dried and ground into a fine powder with a particle size ≤ 0.60 mm. The BLP was stored in a refrigerator in a tightly closed container until analyses.

### 3.2. Pasta Preparation

The pasta was prepared with durum semolina flour, water, olive oil and salt purchased from local stores. The percentage composition of control and fortified pasta is shown in Table 1. BLP was added to the standard control pasta formulation as an additional ingredient in the following concentrations: 0% (C), 2.5% (B2.5) and 5% (B5). The ingredients were mixed for 5 min in an electric pasta maker (Ariete Pastamatic 1581, Florence, Italy) and extruded through a penne-forming die with the same equipment. 

Both fresh and cooked pasta samples were freeze-dried, ground into a fine powder with a particle size ≤ 0.60 mm and stored in a refrigerator in a tightly closed container until chemical and colour analyses.

### 3.3. Cooking Properties

The cooking properties were determined according to the American Association of Cereal Chemists Official Methods [46]. To evaluate cooking properties, 20 g of pasta was cooked in 300 mL of boiling distilled water.

#### 3.3.1. Optimal Cooking Time

OCT (or *al dente* point) is defined as the time required to observe the disappearance of the starchy white core, indicating that the starch at the centre has gelatinized in pasta manually squeezed between two glass plates. 

#### 3.3.2. Cooking Loss

Cooking loss indicates the amount of dry matter lost in the cooking water. Cooking water was collected in a beaker and dried in an air oven at 105 °C until completely evaporated. Cooking loss was calculated according to the following equation to evaluate the presence of dry matter from pasta and expressed as a percentage of the mass of the starting material.

$$\mathrm{Cooking}\;\mathrm{loss}=\left(\frac{\mathrm{mass}\;\mathrm{of}\;\mathrm{dry}\;\mathrm{residue}\;\mathrm{in}\;\mathrm{cooking}\;\mathrm{water}}{\mathrm{mass}\;\mathrm{of}\;\mathrm{uncooked}\;\mathrm{pasta}}\right)\times 100\%$$

#### 3.3.3. Water Absorption

Water absorption was determined as the percentage of weight increase in relation to uncooked pasta and was calculated according to the following equation:

$$\mathrm{Water}\;\mathrm{absorption}=\left(\frac{\mathrm{mass}\;\mathrm{ofcooked}\;\mathrm{pasta}-\mathrm{mass}\;\mathrm{of}\;\mathrm{uncooked}\;\mathrm{pasta}}{\mathrm{mass}\;\mathrm{of}\;\mathrm{uncooked}\;\mathrm{pasta}}\right)\times 100\%$$

#### 3.3.4. Swelling Index

The SI was determined by drying a cooked pasta sample to constant weight in a drying oven for 24 h and calculated according to the following equation:

(1) $$\mathrm{SI}=\left(\frac{\mathrm{mass}\;\mathrm{ofcooked}\;\mathrm{pasta}-\mathrm{mass}\;\mathrm{of}\;\mathrm{cooked}\;\mathrm{pasta}\;\mathrm{after}\;\mathrm{drying}}{\mathrm{mass}\;\mathrm{of}\;\mathrm{cooked}\;\mathrm{pasta}\;\mathrm{after}\;\mathrm{drying}}\right)\times 100\%$$

### 3.4. Textural Properties

Textural properties of BLP-fortified pasta were assessed after cooking the pasta at OCT. Textural parameters of firmness and TSF were determined using a TA.HD Plus texture analyser (Stable Micro Systems Ltd., Godalming, UK) equipped with a 5 kg load cell. The pasta samples were compressed at a constant rate of 1.0 mm/s.

### 3.5. Colour Analysis

The colour of freeze-dried and finely ground pasta samples was evaluated using a HunterLab ColorFlex instrument (Hunter Associates Laboratory, Inc., Reston, VA, USA). The colour was expressed in accordance with the CIELab system and the parameters were determined as follows: lightness: L* = 0 (black) − 100 (white); chromatic components: a* = –a* (greenness) – + a* (redness); and b* = –b* (blueness) – + b* (yellowness). Values are presented as the mean of at least nine replicates. The whiteness index (WI) and the browning index (BI) were calculated according to the following equations:

$$\mathrm{WI}\;=100-\sqrt{{\left(100-L^{*}\right)}^{2}+a^{*}{}^{2}+b^{*}{}^{2}}$$

$$\mathrm{BI}\;=\frac{100\cdot \left(x-0.31\right)}{0.17}$$

where

$$x=\frac{a^{*}+1.75L^{*}}{5.645L^{*}+a^{*}-3.012b^{*}}$$

### 3.6. Proximate Composition

The proximate composition was determined using standard methods in freeze-dried samples [47]. Mineral content (ash) was determined using the gravimetric method by burning in a muffle furnace for 1 h at 585 °C (AOAC 923.03). The protein content was analysed using the Kjeldahl method (N ×6.25 for nitrogen to protein conversion) (AOAC 979.09). Finally, the fat content was determined using Soxhlet extraction with hexane (AOAC 923.03). The total carbohydrate content was estimated by subtracting the protein, fat and ash content from 100%.

### 3.7. Sensory Analysis

The sensory quality of products was evaluated by the QDA method according to ISO standard [PN-EN ISO 13299:2016]. In terms of procedure, a list of all descriptors of smell, appearance, taste and texture was prepared (Table S1). All attributes were defined to equal understanding by assessors. A 10 centimetre scale with arbitrary units was used for the evaluation of each attribute. All scales of odour and taste had edge definitions, which were “not intensive–very intensive”. Additionally, the overall quality of the investigated products was estimated as the summary of all evaluated attributes. The edge definitions for a given attribute were “poor quality–very good quality”.

Assessment of the products was carried out by a previously sensory panel (6 persons), who had been trained and monitored according to ISO guidelines [PN-EN ISO 8586: 2014]. Assessments were carried out in a sensory laboratory room, which fulfils the requirements of the ISO standards [PN-EN ISO 8589:2010]. A computerised sensory program, FIZZ (Biosystemes, Counternon, France), was used for analysis and graphical presentation of the collected results.

### 3.8. Volatile Organic Compounds Analysis

VOCs were extracted using the SPME method described in [38], with slight modifications. Briefly, 4 g of sample pasta cooked at OCT was placed in 20 mL headspace vials closed with an aluminium crimp cap and a silicone/PTFE septum. Extraction was manually performed using Divinylbenzene/Carboxen/Polydimethylsiloxane (DVB/CAR/PDMS) fibre (Supelco, Bellefonte, PA, USA) and a thermomixer (MultiTherm shaker, Benchmark Scientific, Edison, NJ, USA). Samples were equilibrated for 5 min at 60 °C at 500 rpm, and the fibre was exposed for 30 min at 60 °C at 500 rpm. The VOCs were desorbed for 10 min with the injector port set at 250 °C in a splitless mode in a 7890 A gas chromatograph coupled with a 5975 C mass-selective detector (Agilent Technologies, Santa Clara, CA, USA). VOCs were separated using a capillary SupelcoWAX 10 column (30 m length, 250 μm internal diameter, 0.25 μm film thickness, Supelco, Bellefonte, PA, USA). The oven temperature was initially set at 40 °C for 2 min, then increased to 240 °C at 6 °C/min and held for 2 min. Helium was used as carrier gas at a constant flow rate of 1 mL/min. The detector was operated in electron impact (EI) ionization mode at 70 eV. The scan range was from 35 to 550. The ion source temperature was at 240 °C.

Compounds were tentatively identified by comparison of the mass spectra with the NIST/EPA/NIH Mass Spectral Library (version 2.2, 2014, Gaithersburg, MD, USA) for records with a matching score above 70% and confirmed by comparing the Kovats retention indices. For the detected compounds, the odour descriptions were assigned based on the online database [37].

### 3.9. Statistical Analysis

All chemical and technological analyses were performed in triplicate. The results were subjected to a one-way analysis of variance (ANOVA) with Fisher’s LSD as a post hoc test using IBM^®^ SPSS™ Statistics 27 (Armonk, NY, USA) and STATISTICA version 13.3 software (Statsoft, Tulsa, OK, USA). The significance of differences between the samples was set at a *p*-value < 0.05. To identify the differences between the VOC distribution between the BLP and pasta products, as well as the differences between pasta products based on VOC and sensory analyses, orthogonal partial least squares discriminant analysis (OPLS-DA) was used. To assess the contribution of each variable to the model and group separation, variable importance in projection (VIP) scores were measured. Multivariate analysis was performed using SIMCA 16 software (Umetrics, Umeå, Sweden).

## 4. Conclusions

In this study, the suitability of BLP as an ingredient in the manufacture of fortified wheat pasta was investigated based on analysis of the technological, nutritional and sensory properties of the developed product. The obtained results indicate that BLP can be successfully used as an additive in pasta products. Incorporation of BLP enhanced the mineral content of pasta and improved its appearance without compromising the cooking, texture and sensory features. Addition of up to 5% of BLP into pasta products was found not to deteriorate the quality of durum pasta nor its sensory quality, which was rated as high. However, as an increase in cabbage-like aroma and the content of VOCs was noted with increased BLP concentration, the concentration of BLP additive cannot be too high.

In summary, BLP-enriched pasta can be used to increase vegetable consumption in the general public without substantial changes in the daily diet. Moreover, the utilization of broccoli leaves in new product development could facilitate the management of vegetable processing waste.

## Figures and Tables

**Table 1:** Formulations of control and fortified pasta. C (control pasta); B2.5 pasta fortified with 2.5% broccoli leaf powder; B5 (pasta fortified with 5% broccoli leaf powder).

	C	B2.5	B5
Semolina [g]	400.0	400.0	400.0
Water [g]	140.0	140.0	140.0
Olive oil [g]	15.0	15.0	15.0
Salt [g]	2.0	2.0	2.0
BLP [g]	0.0	10.0	20.0

**Table 2:** Proximate composition, colour characteristics, cooking and texture properties of BLP and experimental pasta expressed as mean ± standard deviation (SD).

	BLP	C	B2.5	B5
Moisture (%)	-	61.04 ± 0.26 ^a^*	63.64 ± 0.01 ^b^	60.75 ± 0.15 ^a^
Ash (%)	10.94 ± 0.04	0.88 ± 0.01 ^a^	1.00 ± 0.03 ^b^	1.19 ± 0.01 ^c^
Protein (%)	25.66 ± 0.27	13.06 ± 0.21 ^a^	13.17 ± 0.21 ^a^	13.26 ± 0.07 ^a^
Fat (%)	3.94 ± 0.21	3.94 ± 0.15 ^a^	4.79 ± 0.04 ^c^	4.45 ± 0.10 ^b^
Carbohydrate (%)	59.46 ± 0.07	21.08 ± 0.5 ^a^	17.41 ± 0.18 ^b^	20.23 ± 0,16 ^a^
L*	46.18 ± 0.03	87.51 ± 0.01 ^a^	76.05 ± 0.03 ^b^	67.61 ± 0.02 ^c^
A*	−9.33 ± 0.03	−0.12 ± 0.01 ^a^	−6.25 ± 0.02 ^b^	−6.26 ± 0.02 ^b^
B*	27.39 ± 0.06	14.05 ± 0.02 ^a^	28.14 ± 0.03 ^b^	31.48 ± 0.02 ^c^
WI	38.89 ± 0.05	81.20 ± 0.02 ^a^	62.52 ± 0.03 ^b^	54.41 ± 0.02 ^c^
BI	66.67 ± 0.29	16.99 ± 0.02 ^a^	38.20 ± 0.07 ^b^	52.57 ± 0.06 ^c^
OCT (min)	-	3.56 ± 0.08 ^a^	3.03 ± 0.04 ^b^	3.44 ± 0.10 ^a^
Cooking loss (%)	-	5.96 ± 0.38 ^b^	7.47 ± 0.19 ^a^	5.95 ± 0.05 ^b^
Water absorption (%)	-	62.26 ± 1.36 ^a^	55.55 ± 5.65 ^a^	60.45 ± 3.88 ^a^
Swelling index (%)	-	0.82 ± 0.04 ^b^	1.08 ± 0.05 ^a^	1.07 ± 0.04 ^a^
Firmness (kg)	-	13.37 ± 0.17 ^a^	12.09 ± 0.10 ^b^	11.96 ± 0.24 ^b^
TSF (kg s^−1^)	-	87.30 ± 4.11 ^a^	76.09 ± 1.52 ^b^	73.90 ± 5.53 ^b^

(*) Different letters in the same line indicate a significant difference (*p* < 0.05). C: control pasta; B2.5: pasta fortified with 2.5% broccoli leaf powder; B5: pasta fortified with 5% broccoli leaf powder; WI: whiteness index; BI: browning index; OCT: optimal cooking time; TSF: total shearing force.

**Table 3:** VOCs of previously reported aroma activity identified in broccoli leaf powder (BLP), control (C) and experimental pasta (B2.5; B5). Data are expressed as the peak area [×10^6^]. Statistical analysis was performed to compare pasta samples (C, B2.5 and B5); different letters in a row indicate statistically significant (*p*-value < 0.05) differences between the samples.

Volatile Compound	RI_Exp_ *	RI_Lit_ **	BLP	C	B2.5	B5	Odour Description ***
*Aldehydes*							
Propanal	817	801	4.85 ± 0.45	ND ****	5.17 ± 0.55 ^a^*****	5.89 ± 0.25 ^a^	fresh, fruity, malty
Butanal	891	883	2.01 ± 0.13	ND	ND	0.40 ± 0.04	malty, sweaty
2-Methylbutanal	922	915	5.48 ± 0.42	ND	0.19 ± 0.02 ^b^	0.30 ± 0.03 ^a^	malty
3-Methylbutanal	927	920	7.87 ± 0.49	ND	0.41 ± 0.05 ^a^	0.54 ± 0.09 ^a^	malty
Pentanal	987	983	ND	2.22 ± 0.19 ^b^	2.74 ± 0.15 ^a^	2.98 ± 0.08 ^a^	green, fatty, mouldy
Hexanal	1087	1093	50.83 ± 0.98	53.66 ± 4.91 ^a^	31.91 ± 1.53 ^b^	28.05 ± 1.98 ^b^	green grass, fatty
(*E*)-2-Methyl-2-butenal	1097	1098	12.81 ± 1.15	ND	0.53 ± 0.28 ^b^	2.14 ± 0.31 ^a^	green, fruity, aromatic
(*E*)-2-Pentenal	1139	1140	20.12 ± 0.2	0.13 ± 0.00 ^c^	1.49 ± 0.11 ^b^	2.24 ± 0.07 ^a^	oily, fatty, fruity
Heptanal	1189	1188	ND	5.29 ± 0.39 ^a^	3.97 ± 0.61 ^b^	4.78 ± 0.15 ^a, b^	fatty, citrus-like
(*E*)-2-Hexenal	1227	1220	30.85 ± 0.53	1.38 ± 0.11 ^c^	4.01 ± 0.22 ^b^	5.28 ± 0.21 ^a^	green apple-like, bitter almond-like
(*Z*)-4-Heptenal	1251	1235	13.92 ± 0.07	ND	0.87 ± 0.24 ^b^	1.45 ± 0.16 ^a^	fish-like, train oil-like
Octanal	1293	1291	3.38 ± 0.11	1.73 ± 0.29 ^b^	5.48 ± 0.62 ^a^	4.63 ± 0.40 ^a^	citrus-like, green
Nonanal	1398	1390	7.47 ± 0.13	2.39 ± 0.33 ^b^	4.01 ± 0.29 ^a^	4.88 ± 0.65 ^a^	citrus-like, soapy
(*E*)-2-Octenal	1440	1437	2.76 ± 0.1	3.01 ± 0.18 ^b^	6.59 ± 0.66 ^a^	6.52 ± 0.77 ^a^	fatty, nutty
(*E,E*)-2,4-Heptadienal	1477	1497	26.15 ± 0.47	ND	1.04 ± 0.11 ^b^	1.65 ± 0.18 ^a^	green, fatty, flowery
Benzaldehyde	1538	1530	37.59 ± 0.92	2.26 ± 0.94 ^a^	1.32 ± 0.10 ^a^	1.95 ± 0.34 ^a^	bitter almond-like, marzipan-like
(*E*)-2-Nonenal	1547	1543	ND	1.96 ± 0.15 ^c^	3.78 ± 0.45 ^b^	5.10 ± 0.48 ^a^	fatty
(*E,Z*)-2,6-Nonadienal	1598	1596	2.05 ± 0.71	ND	0.26 ± 0.04 ^b^	0.55 ± 0.06 ^a^	green, cucumber-like
Safranal	1661	1648	4.71 ± 0.78	ND	0.36 ± 0.06 ^b^	0.53 ± 0.04 ^a^	herbal
(*E,E*)-2,4-Nonadienal	1716	1691	ND	0.29 ± 0.03 ^a^	0.11 ± 0.04 ^b^	0.10 ± 0.04 ^b^	fatty, green
(*E,E*)-2,4-Decadienal	1779	1786	ND	0.64 ± 0.07 ^a^	0.88 ± 0.07 ^a^	0.84 ± 0.09 ^a^	fatty, deep-fried
*Ketones*							
Acetone	838	813	8.08 ± 0.32	ND	0.30 ± 0.09	ND	pungent, sweet
2-Butanone	909	905	1.31 ± 0.3	ND	0.49 ± 0.05 ^a^	0.26 ± 0.04 ^b^	ethereal, fruity
1-Penten-3-one	1030	1024	2.23 ± 0.12	ND	0.17 ± 0.09 ^a^	0.25 ± 0.06 ^a^	green, pungent, mustard
3-Penten-2-one	1136	1123	5.75 ± 0.08	ND	ND	ND	fruity, pungent
4-Methyl-2-hexanone	1188	NA	3.41 ± 0.33	0.28 ± 0.06 ^b^	0.75 ± 0.15 ^a^	0.35 ± 0.06 ^b^	ethereal, bitter almond-like
6-Methyl-2-heptanone	1246	1228	6.19 ± 0.23	ND	0.61 ± 0.11 ^b^	1.16 ± 0.06 ^a^	fruity, sour
2-Octanone	1291	1287	0.87 ± 0.04	ND	0.33 ± 0.08	ND	soapy, fruity
6-Methyl-5-hepten-2-one	1348	1341	88.61 ± 0.55	0.85 ± 0.11 ^c^	6.31 ± 0.83 ^b^	9.63 ± 0.46 ^a^	citrus
3,5,5-Trimethyl-2-cyclohexen-1-one	1413	1544	10.77 ± 0.16	ND	1.39 ± 0.35 ^b^	1.96 ± 0.28 ^a^	woody
3-Octen-2-one	1416	1408	ND	1.06 ± 0.12 ^a^	1.73 ± 0.05 ^a^	1.26 ± 0.26 ^a^	flowery, spicy
(E,E)-3,5-octadien-2-one	1531	1569	188.13 ± 4.64	ND	2.22 ± 0.3 ^b^	4.42 ± 0.32 ^a^	fruity, musty, fatty
4-Acetyl-1-methylcyclohexene	1568	1570	3.26 ± 0.39	ND	ND	ND	seasoning
(E)-6-Methyl-3,5-heptadien-2-one	1606	1582	11.24 ± 0.86	ND	0.43 ± 0.07 ^a^	0.73 ± 0.17 ^a^	spicy
Acetophenone	1668	1660	2.76 ± 0.69	0.29 ± 0.05 ^a^	0.22 ± 0.01 ^a^	0.30 ± 0.09 ^a^	fruity, foxy, bitter almond-like, rubber-like
6,10-Dimethyl-5,9-undecadien-2-one	1865	1867	10.57 ± 0.51	ND	0.67 ± 0.07 ^b^	1.23 ± 0.16 ^a^	floral
*Alcohols*							
Ethanol	944	934	2.26 ± 0.12	ND	ND	ND	alcoholic
1-Butanol	1152	1125	19.18 ± 0.38	ND	0.24 ± 0.12 ^b^	0.42 ± 0.04 ^a^	malty
1-Penten-3-ol	1167	1175	105.58 ± 2.07	ND	3.00 ± 0.18 ^a^	0.77 ± 0.11 ^b^	pungent, milk-like
1-Pentanol	1256	1252	10.77 ± 0.24	3.38 ± 0.46 ^a^	3.01 ± 0.3 ^a,b^	2.24 ± 0.21 ^b^	fruity, ethereal
(*E*)-2-Penten-1-ol	1317	1313	3.88 ± 0.25	ND	ND	ND	green
(*Z*)-2-Penten-1-ol	1326	1323	69.57 ± 0.76	ND	1.16 ± 0.17	ND	musty, compost-like
1-Hexanol	1358	1360	ND	14.63 ± 2.09 ^a,b^	15.00 ± 1.47 ^a^	11.38 ± 0.05 ^b^	herbal, grassy, marzipan-like
(*Z*)-3-Hexen-1-ol	1369	1386	ND	0.38 ± 0.03 ^b^	0.96 ± 0.09 ^a^	1.01 ± 0.04 ^a^	green
1-Octen-3-ol	1455	1446	15.77 ± 0.17	3.74 ± 0.48 ^b^	14.45 ± 2.81 ^a^	13.16 ± 1.52 ^a^	earthy, mushroom-like
Heptanol	1459	1456	ND	1.12 ± 0.09 ^a^	1.05 ± 0.11 ^a^	1.02 ± 0.02 ^a^	fruity, soapy
3-Octanol	1472	1423	ND	0.17 ± 0.02 ^a^	0.18 ± 0.02 ^a^	0.16 ± 0.02 ^a^	citrus-like, soapy
2-Ethyl-1-Hexanol	1492	1490	2.66 ± 0.06	1.57 ± 0.17 ^a^	1.58 ± 0.18 ^a^	1.67 ± 0.06 ^a^	ethereal, fruity
(*E*)-2-Octen-1-ol	1621	1618	ND	0.41 ± 0.08 ^b^	4.30 ± 1.01 ^a^	4.17 ± 0.74 ^b^	soapy
1-Nonanol	1664	1665	ND	0.48 ± 0.03 ^a^	0.66 ± 0.13 ^a^	0.57 ± 0.02 ^a^	soapy, fruity
*Sulphur compounds*							
Dimethyl sulphide	715	720	4.62 ± 0.36	ND	0.42 ± 0.03 ^a^	0.41 ± 0.03 ^a^	cabbage-like, sulphur
Dimethyl sulphoxide	1587	1584	5.2 ± 1.03	ND	ND	ND	alliaceous
*Terpenes*							
Limonene	1198	1190	24.94 ± 1.12	13.04 ± 0.72 ^a^	10.35 ± 1.24 ^b^	12.69 ± 0.42 ^a,b^	citrus-like
Linalool	1553	1545	ND	0.28 ± 0.05 ^c^	1.26 ± 0.13 ^b^	2.17 ± 0.06 ^a^	citrus-like, flowery
Beta-cyclocitral	1637	1617	51.08 ± 2.21	ND	1.63 ± 0.22 ^b^	3.45 ± 0.14 ^a^	tropical, fruity
β-ionone	1955	1898	34.98 ± 0.64	ND	0.99 ± 0.08 ^b^	2.16 ± 0.17 ^a^	floral
β-ionone-5,6-epoxide	2011	2008	27.37 ± 0.33	ND	0.74 ± 0.10 ^b^	1.61 ± 0.21 ^a^	fruity
Dihydroactinidiolide	2385	2332	35.45 ± 1.65	ND	0.55 ± 0.06 ^b^	1.35 ± 0.23 ^a^	fruity
*Alkanes*							
Dodecane	1194	1227	6.51 ± 0.13	ND	ND	ND	alkane
Tetradecane	1396	1400	11.93 ± 1.25	ND	ND	ND	waxy
*Furan derivatives*							
2-ethylfuran	962	955	2.41 ± 0.37	ND	0.43 ± 0.06 ^b^	0.69 ± 0.21 ^a^	sweet, burnt, earthy, malty
2-pentylfuran	1234	1249	1.96 ± 0.33	5.52 ± 0.84 ^a^	1.02 ± 0.31 ^b^	2.23 ± 0.32 ^b^	fruity
*cis*-Linalool oxide	1451	1513	7.48 ± 0.25	ND	0.08 ± 0.04 ^b^	0.20 ± 0.02 ^a^	earthy
5-Ethyl-2(5H)-furanone	1781	1757	4.04 ± 0.38	ND	0.16 ± 0.04 ^a^	0.15 ± 0.03 ^a^	sweet, spicy
*Miscellaneous*							
Styrene	1264	1273	ND	0.38 ± 0.03 ^a^	0.63 ± 0.16 ^a^	0.62 ± 0.12 ^a^	balsamic
4-(2-Propenyl)-phenol	1748	2342	3.47 ± 0.3	ND	0.19 ± 0.05 ^a^	0.34 ± 0.06 ^a^	sweet, burned
**Total peak area**			**1038.02 ± 6.67**	**122.51 ± 9.33 ^c^**	**140.97 ± 9.85 ^b^**	**162.4.16 ± 4.16 ^a^**	

(*) Experimental retention index; (**) retention index from literature; (***) odour descriptions adapted from the online database; (****) not detected; (*****) Different letters in the same line indicate a significant difference (*p* < 0.05).

## Data Availability

Data are contained within the article or Supplementary Material.

## Supplementary Materials

The following supporting information can be downloaded at: https://www.mdpi.com/article/10.3390/molecules27154672/s1, Table S1: Sensory attributes, their definitions and scale edges used in QDA of pasta products.; Figure S1: Volatile organic compounds associated with BLP and experimental pasta.

## Sample Availability

Samples of the compounds are not available from the authors.

## References

[1] Oliviero T., Fogliano V. (2016). Food Design Strategies to Increase Vegetable Intake: The Case of Vegetable Enriched Pasta. Trends Food Sci. Technol..

[2] Folkvord F., Naderer B., Coates A., Boyland E. (2021). Promoting Fruit and Vegetable Consumption for Childhood Obesity Prevention. Nutrients.

[3] Silva E., Gerritsen L., Dekker M., van der Linden E., Scholten E. (2013). High Amounts of Broccoli in Pasta-like Products: Nutritional Evaluation and Sensory Acceptability. Food Funct..

[4] Petitot M., Boyer L., Minier C., Micard V. (2010). Fortification of Pasta with Split Pea and Faba Bean Flours: Pasta Processing and Quality Evaluation. Food Res. Int..

[5] Bustos M.C., Perez G.T., Leon A.E. (2015). Structure and Quality of Pasta Enriched with Functional Ingredients. RSC Adv..

[6] Nilusha R.A.T., Jayasinghe J.M.J.K., Perera O.D.A.N., Perera P.I.P. (2019). Development of Pasta Products with Nonconventional Ingredients and Their Effect on Selected Quality Characteristics: A Brief Overview. Int. J. Food Sci..

[7] Bruno J.A., Konas D.W., Matthews E.L., Feldman C.H., Pinsley K.M., Kerrihard A.L. (2019). Sprouted and Non-Sprouted Chickpea Flours: Effects on Sensory Traits in Pasta and Antioxidant Capacity. Pol. J. Food Nutr. Sci..

[8] Padalino L., Mastromatteo M., Lecce L., Cozzolino F., del Nobile M.A. (2013). Manufacture and Characterization of Gluten-Free Spaghetti Enriched with Vegetable Flour. J. Cereal Sci..

[9] Lorusso A., Verni M., Montemurro M., Coda R., Gobbetti M., Rizzello C.G. (2017). Use of Fermented Quinoa Flour for Pasta Making and Evaluation of the Technological and Nutritional Features. LWT.

[10] Desai A.S., Brennan M.A., Brennan C.S. (2018). Effect of Fortification with Fish (*Pseudophycis bachus*) Powder on Nutritional Quality of Durum Wheat Pasta. Foods.

[11] Michalak-Majewska M., Teterycz D., Muszyński S., Radzki W., Sykut-Domańska E. (2020). Influence of Onion Skin Powder on Nutritional and Quality Attributes of Wheat Pasta. PLoS ONE.

[12] Gómez M., Martinez M.M. (2018). Fruit and Vegetable By-Products as Novel Ingredients to Improve the Nutritional Quality of Baked Goods. Crit. Rev. Food Sci. Nutr..

[13] Domínguez-Perles R., Martínez-Ballesta M.C., Carvajal M., García-Viguera C., Moreno D.A. (2010). Broccoli-Derived by-Products—A Promising Source of Bioactive Ingredients. J. Food Sci..

[14] Reguengo L.M., Salgaço M.K., Sivieri K., Maróstica Júnior M.R. (2022). Agro-Industrial by-Products: Valuable Sources of Bioactive Compounds. Food Res. Int..

[15] Orak H.H., Bahrisefit I.S., Sabudak T. (2019). Antioxidant Activity of Extracts of Soursop (*Annona muricata* L.) Leaves, Fruit Pulps, Peels, and Seeds. Pol. J. Food Nutr. Sci..

[16] Savić A., Alimpić Aradski A., Živković J., Šavikin K., Jarić S., Marin P.D., Duletić-Laušević S. (2021). Phenolic Composition, and Antioxidant and Antineurodegenerative Potential of Methanolic Extracts of Fruit Peel and Flesh of Pear Varieties from Serbia. Pol. J. Food Nutr. Sci..

[17] Guglielmetti A., Fernandez-Gomez B., Zeppa G., del Castillo M.D. (2019). Nutritional Quality, Potential Health Promoting Properties and Sensory Perception of an Improved Gluten-Free Bread Formulation Containing Inulin, Rice Protein and Bioactive Compounds Extracted from Coffee Byproducts. Pol. J. Food Nutr. Sci..

[18] Karwacka M., Gumkowska M., Rybak K., Ciurzyńska A., Janowicz M. (2021). Impact of Sodium Alginate and Dried Apple Pomace Powder as a Carrier Agent on the Properties of Freeze-Dried Vegetable Snacks. Pol. J. Food Nutr. Sci..

[19] Liu M., Zhang L., Ser S.L., Cumming J.R., Ku K.-M. (2018). Comparative Phytonutrient Analysis of Broccoli By-Products: The Potentials for Broccoli By-Product Utilization. Molecules.

[20] Drabińska N., Ciska E., Szmatowicz B., Krupa-Kozak U. (2018). Broccoli By-Products Improve the Nutraceutical Potential of Gluten-Free Mini Sponge Cakes. Food Chem..

[21] Drabińska N. (2022). The Evaluation of Amino Acid Profiles in Gluten-Free Mini Sponge Cakes Fortified with Broccoli By-Product. Separations.

[22] Li H., Xia Y., Liu H.-Y., Guo H., He X.-Q., Liu Y., Wu D.-T., Mai Y.-H., Li H.-B., Zou L. (2022). Nutritional Values, Beneficial Effects, and Food Applications of Broccoli (*Brassica oleracea* Var. *Italica plenck*). Trends Food Sci. Technol..

[23] Berndtsson E., Andersson R., Johansson E., Olsson M.E. (2020). Side Streams of Broccoli Leaves: A Climate Smart and Healthy Food Ingredient. Int. J. Environ. Res. Public Health.

[24] Lafarga T., Gallagher E., Bademunt A., Viñas I., Bobo G., Villaró S., Aguiló-Aguayo I. (2019). Bioaccessibility, Physicochemical, Sensorial, and Nutritional Characteristics of Bread Containing Broccoli Co-Products. J. Food Processing Preserv..

[25] Krupa-Kozak U., Drabińska N., Bączek N., Šimková K., Starowicz M., Jeliński T. (2021). Application of Broccoli Leaf Powder in Gluten-Free Bread: An Innovative Approach to Improve Its Bioactive Potential and Technological Quality. Foods.

[26] Dominguez-Perles R., Moreno D.A., Carvajal M., Garcia-Viguera C. (2011). Composition and Antioxidant Capacity of a Novel Beverage Produced with Green Tea and Minimally-Processed Byproducts of Broccoli. Innov. Food Sci. Emerg. Technol..

[27] Campas-Baypoli O.N., Snchez-Machado D.I., Bueno-Solano C., Núñez-Gastélum J.A., Reyes-Moreno C., López-Cervantes J. (2009). Biochemical Composition and Physicochemical Properties of Broccoli Flours. Int. J. Food Sci. Nutr..

[28] Ajila C.M., Aalami M., Leelavathi K., Rao U.J.S.P. (2010). Mango Peel Powder: A Potential Source of Antioxidant and Dietary Fiber in Macaroni Preparations. Innov. Food Sci. Emerg. Technol..

[29] Brennan C.S., Kuri V., Tudorica C.M. (2004). Inulin-Enriched Pasta: Effects on Textural Properties and Starch Degradation. Food Chem..

[30] Stable Micro Systems Cutting and Shearing Testing|Texture Analyser. https://www.stablemicrosystems.com/cutting-and-shearing-testing.html.

[31] Gull A., Prasad K., Kumar P. (2015). Effect of Millet Flours and Carrot Pomace on Cooking Qualities, Color and Texture of Developed Pasta. LWT—Food Sci. Technol..

[32] Ranawana V., Campbell F., Bestwick C., Nicol P., Milne L., Duthie G., Raikos V. (2016). Breads Fortified with Freeze-Dried Vegetables: Quality and Nutritional Attributes. Part II: Breads Not Containing Oil as an Ingredient. Foods.

[33] Özyurt G., Uslu L., Yuvka I., Gökdoğan S., Atci G., Ak B., Işik O. (2015). Evaluation of the Cooking Quality Characteristics of Pasta Enriched with Spirulina Platensis. J. Food Qual..

[34] Cappa C., Alamprese C. (2017). Brewer’s Spent Grain Valorization in Fiber-Enriched Fresh Egg Pasta Production: Modelling and Optimization Study. LWT—Food Sci. Technol..

[35] Beleggia R., Platani C., Spano G., Monteleone M., Cattivelli L. (2009). Metabolic Profiling and Analysis of Volatile Composition of Durum Wheat Semolina and Pasta. J. Cereal Sci..

[36] de Flaviis R., Sacchetti G., Mastrocola D. (2021). Wheat Classification According to Its Origin by an Implemented Volatile Organic Compounds Analysis. Food Chem..

[37] Kreissl J., Mall V., Steinhaus P., Steinhaus M. Leibniz-LSB@TUM Odorant Database, Version 1.0. https://www.leibniz-lsb.de/en/databases/leibniz-lsbtum-odorant-database.

[38] Wieczorek M.N., Jeleń H.H. (2019). Volatile Compounds of Selected Raw and Cooked Brassica Vegetables. Molecules.

[39] Marcinkowska M., Frank S., Steinhaus M., Jeleń H.H. (2021). Key Odorants of Raw and Cooked Green Kohlrabi (*Brassica oleracea* Var. *Gongylodes* L.). J. Agric. Food Chem..

[40] Wieczorek M.N., Majcher M., Jeleń H. (2020). Comparison of Three Extraction Techniques for the Determination of Volatile Flavor Components in Broccoli. Foods.

[41] Wieczorek M.N., Majcher M.A., Jeleń H.H. (2021). Identification of Aroma Compounds in Raw and Cooked Broccoli. Flavour Fragr. J..

[42] Van Gemert L.J. (2011). Compilations of Odour Threshold Values in Air, Water and Other Media.

[43] Giannetti V., Boccacci Mariani M., Mannino P., Testani E. (2014). Furosine and Flavour Compounds in Durum Wheat Pasta Produced under Different Manufacturing Conditions: Multivariate Chemometric Characterization. LWT—Food Sci. Technol..

[44] Wieczorek M.N., Zhou W., Pawliszyn J. (2022). Sequential Thin Film-Solid Phase Microextraction as a New Strategy for Addressing Displacement and Saturation Effects in Food Analysis. Food Chem..

[45] Gaggiotti S., Shkembi B., Sacchetti G., Compagnone D. (2019). Study on Volatile Markers of Pasta Quality Using GC-MS and a Peptide Based Gas Sensor Array. LWT.

[46] (2000). AACC Approved Methods of Analysis.

[47] Association of Official Analytical Chemists (2005). AOAC Official Methods of Analysis.

