# Supplementary material for: Valorisation of Broccoli By-Products: Technological, Sensory and Flavour Properties of Durum Pasta Fortified with Broccoli Leaf Powder
Source: Molecules. 2022 Jul 22;27(15):4672. doi: 10.3390/molecules27154672 (PMC9332216; doi:10.3390/molecules27154672)
Supplement: Supplementary file 1 [file molecules-27-04672-s001.zip › molecules-1825558-supplementary.pdf]

SUPPLEMENTARY MATERIALS

Valorisation of broccoli by-products: technological, sensory and flavour properties of durum pasta fortified with broccoli leaf powder

Natalia Drabińska[1], Mariana Nogueira[1,2], Beata Szmatowicz[3]

[1]*Department of Chemistry and Biodynamics of Food, Institute of Animal Reproduction and Food Research of Polish Academy of Sciences, Tuwima 10, 10-748 Olsztyn, Poland; n.drabinska@pan.olsztyn.pl*

[2]*Faculty of Biotechnology, Universidade Católica Portuguesa, Porto, Portugal; s-masanogueira@ucp.pt*

[3]*Sensory Laboratory, Institute of Animal Reproduction and Food Research of Polish Academy of Sciences, 10-748 Olsztyn, Poland; b.szmatowicz@pan.olsztyn.pl*

Correspondence: n.drabinska@pan.olsztyn.pl

Table S1. Sensory attributes, their definitions and scale edges used in QDA of pasta products.

Attribute	Definition	Scale edges
Odour		
flour	odour corresponding to wheat flour	none - very intensive
cabbage	odour corresponding to boiled cruciferous vegetables (e.g. broccoli, cabbage)	none - very intensive
Appearance		
creamy	colour intensity (colour intensity according to colour pattern RAL 075 90 20 - scale value 6)	light - dark
green	colour intensity (colour intensity according to colour pattern RAL 110 50 20 - scale value 8)	light - dark
Texture (manual)		
elasticity	the extent to which a piece of product returns to its original length when stretched	low - high
Texture (in the mouth)		
hardness	the force required to bite through the sample placed between the front teeth	low - high
chewiness	the multiplicity of chewing the product to prepare it to swallow	low - high
adhesiveness	degree of adhesiveness while chewing the sample 10 times	low - high
Taste		
flour	as the corresponding odour (measured in the mouth)	none - very intensive
cabbage	as the corresponding odour (measured in the mouth)	none - very intensive
sweet	basic taste illustrated by sucrose dissolved in water (3%)	
aftertaste	lingering sensation after swallowing the sample	none - very intensive
Overall quality	overall quality contains all attributes and their harmonisation	low - high

Figure S1. Volatile organic compounds associated with BLP and experimental pasta.

(BLP)

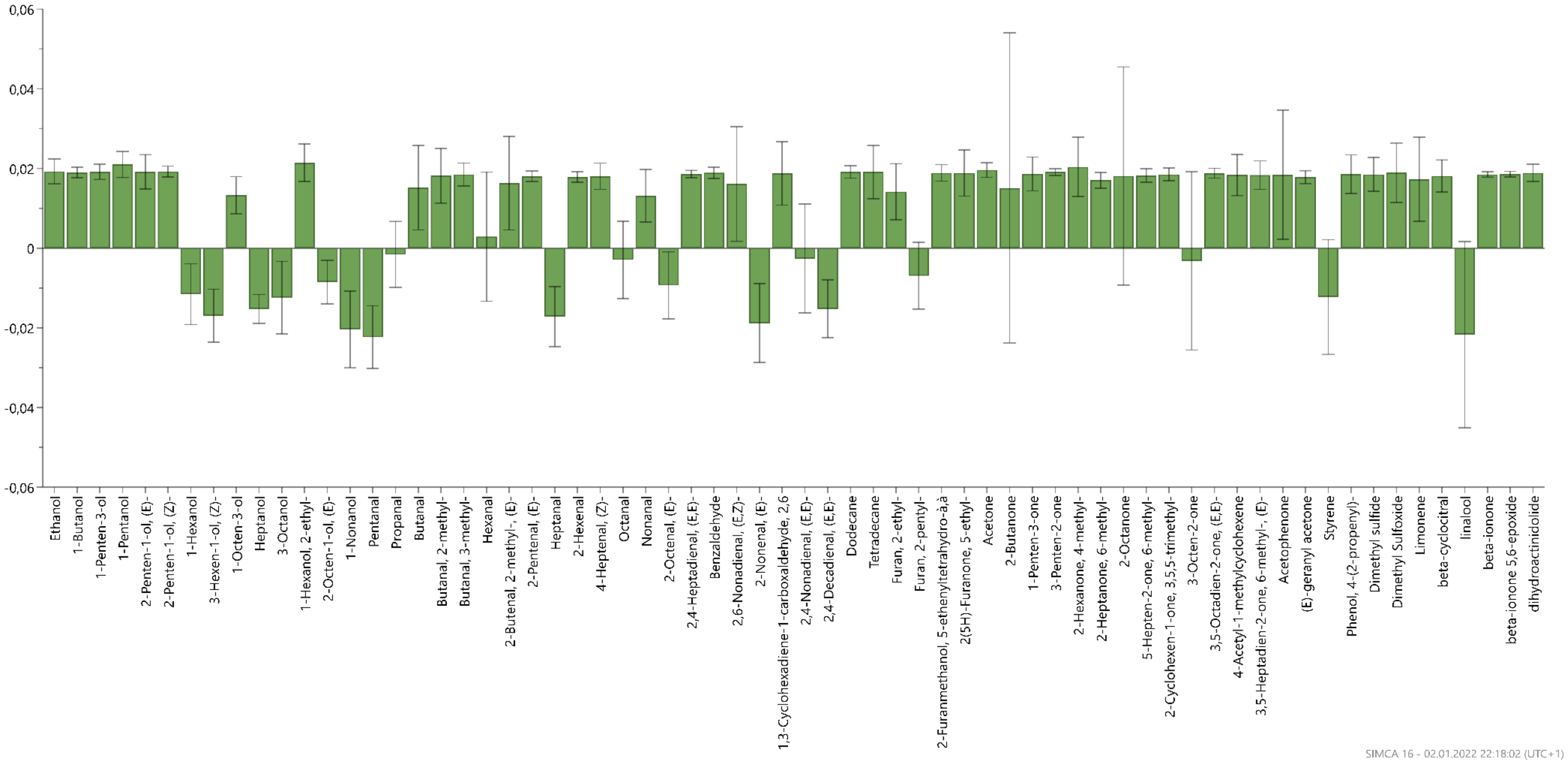

(C)

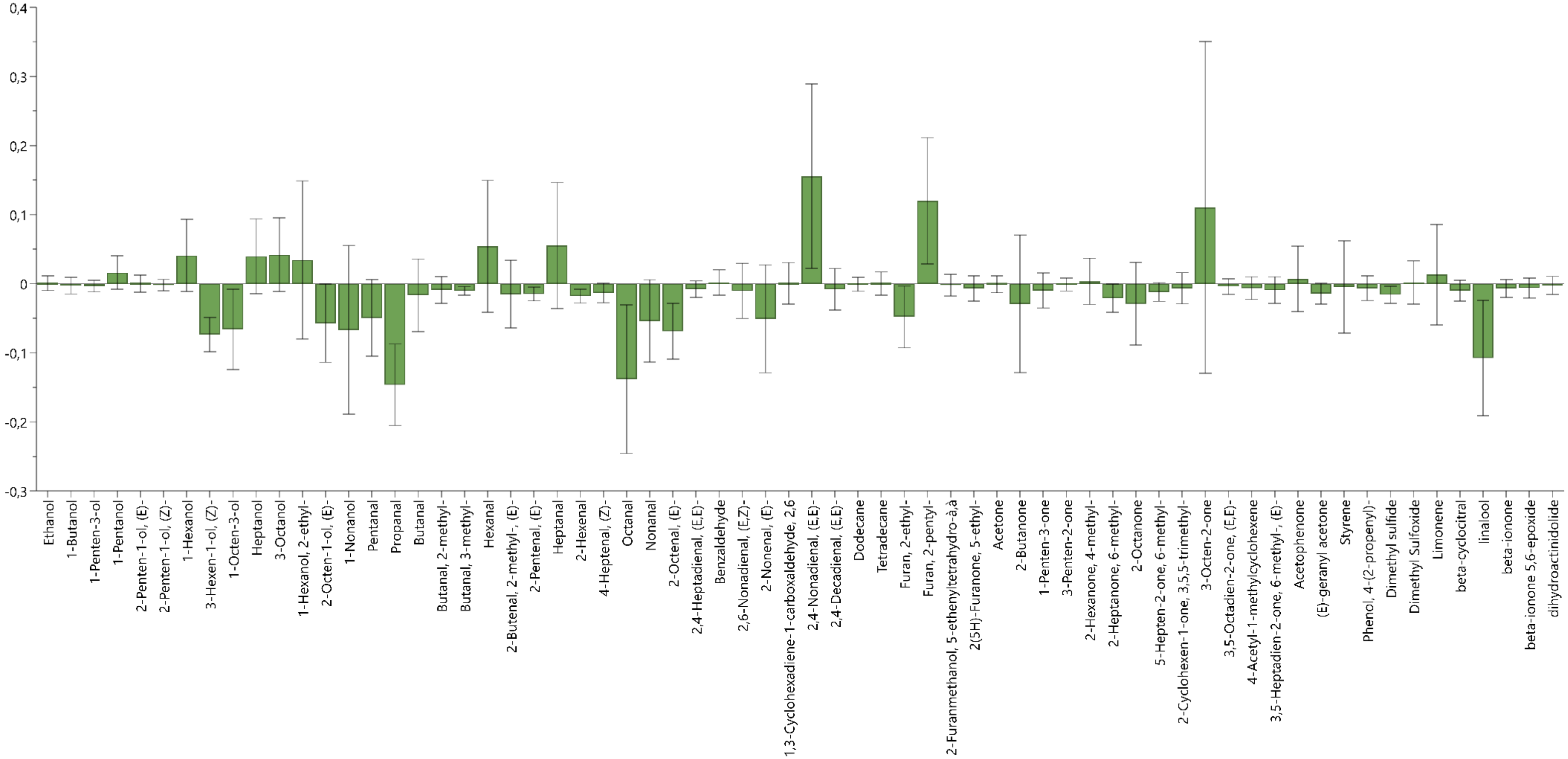
0,4
0,3
0,2
0,1
0
-0,1
-0,2
-0,3
Ethanol
1-Butanol
1-Penten-3-ol
1-Pentanol
2-Penten-1-ol, (E)-
2-Penten-1-ol, (Z)-
1-Hexanol
3-Hexen-1-ol, (Z)-
1-Octen-3-ol
Heptanol
3-Octanol
1-Hexanol, 2-ethyl-
2-Octen-1-ol, (E)-
1-Nonanol
Pentanal
Propanal
Butanal
Butanal, 2-methyl-
Butanal, 3-methyl-
Hexanal
2-Butenal, 2-methyl-, (E)-
2-Pentenal, (E)-
Heptanal
2-Hexenal
4-Heptenal, (Z)-
Octanal
Nonanal
2-Octenal, (E)-
2,4-Heptadienal, (E,E)-
Benzaldehyde
2,6-Nonadienal, (E,Z)-
2-Nonenal, (E)-
1,3-Cyclohexadiene-1-carboxaldehyde, 2,6
2,4-Nonadienal, (E,E)-
2,4-Decadienal, (E,E)-
Dodecane
Tetradecane
Furan, 2-ethyl-
Furan, 2-pentyl-
2-Furanmethanol, 5-ethenyltetrahydro-à,à
2(5H)-Furanone, 5-ethyl-
Acetone
2-Butanone
1-Penten-3-one
3-Penten-2-one
2-Hexanone, 4-methyl-
2-Heptanone, 6-methyl-
2-Octanone
5-Hepten-2-one, 6-methyl-
2-Cyclohexen-1-one, 3,5,5-trimethyl-
3-Octen-2-one
3,5-Octadien-2-one, (E,E)-
4-Acetyl-1-methylcyclohexene
3,5-Heptadien-2-one, 6-methyl-, (E)-
Acetophenone
(E)-geranyl acetone
Styrene
Phenol, 4-(2-propenyl)-
Dimethyl sulfide
Dimethyl Sulfoxide
Limonene
beta-cyclocitral
linalool
beta-ionone
beta-ionone 5,6-epoxide
dihydroactinidolide

(B2.5)

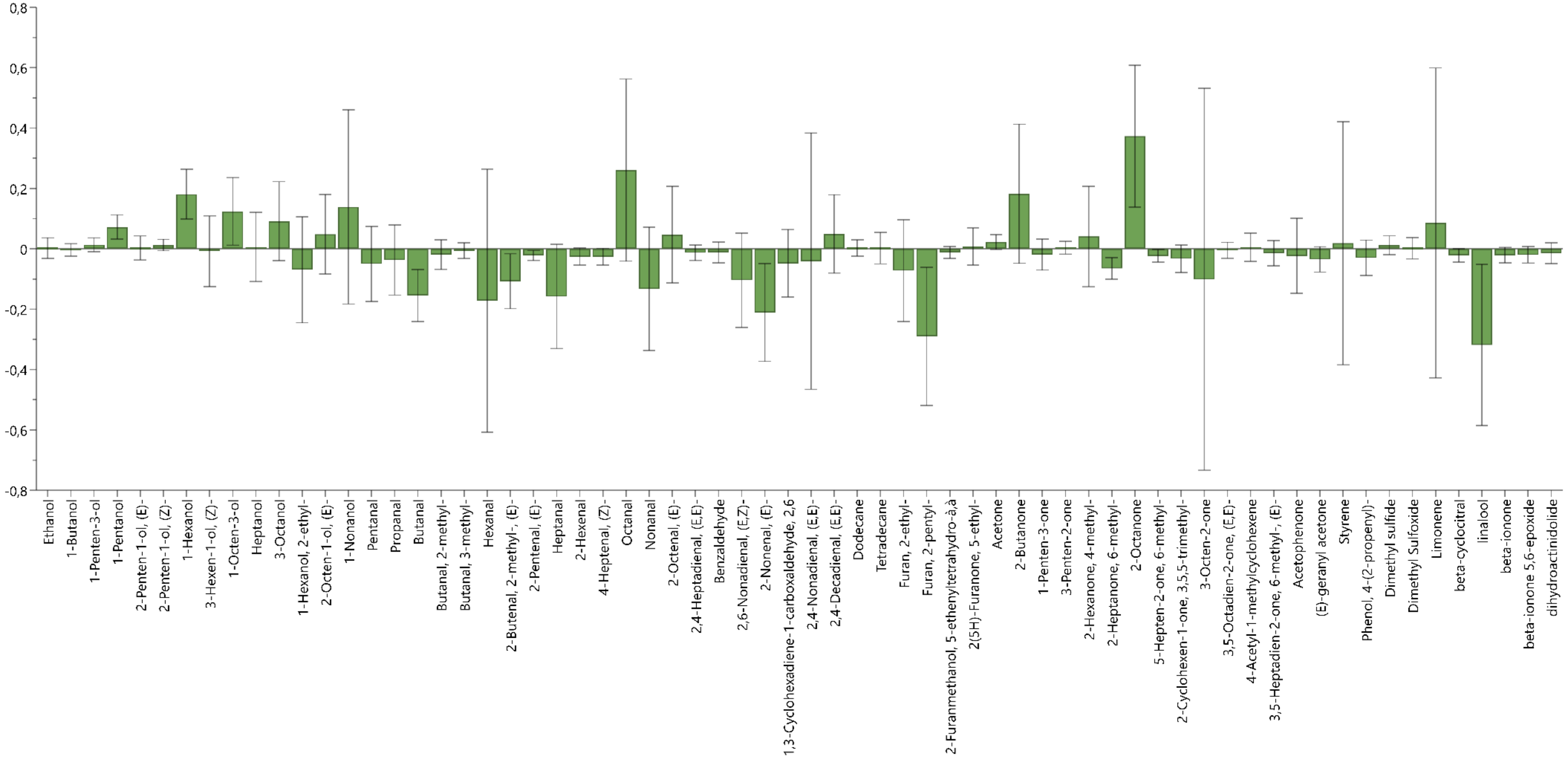
0,8
0,6
0,4
0,2
0
-0,2
-0,4
-0,6
-0,8
Ethanol
1-Butanol
1-Penten-3-ol
1-Pentanol
2-Penten-1-ol, (E)-
2-Penten-1-ol, (Z)-
1-Hexanol
3-Hexen-1-ol, (Z)-
1-Octen-3-ol
Heptanol
3-Octanol
1-Hexanol, 2-ethyl-
2-Octen-1-ol, (E)-
1-Nonanol
Pentanal
Propanal
Butanal
Butanal, 2-methyl-
Butanal, 3-methyl-
Hexanal
2-Butenal, 2-methyl-, (E)-
2-Pentenal, (E)-
Heptanal
2-Hexenal
4-Heptenal, (Z)-
Octanal
Nonanal
2-Octenal, (E)-
2,4-Heptadienal, (E,E)-
Benzaldehyde
2,6-Nonadienal, (E,Z)-
2-Nonenal, (E)-
1,3-Cyclohexadiene-1-carboxaldehyde, 2,6
2,4-Nonadienal, (E,E)-
2,4-Decadienal, (E,E)-
Dodecane
Tetradecane
Furan, 2-ethyl-
Furan, 2-pentyl-
2-Furanmethanol, 5-ethenyltetrahydro-à,à
2(5H)-Furanone, 5-ethyl-
Acetone
2-Butanone
1-Penten-3-one
3-Penten-2-one
2-Hexanone, 4-methyl-
2-Heptanone, 6-methyl-
2-Octanone
5-Hepten-2-one, 6-methyl-
2-Cyclohexen-1-one, 3,5,5-trimethyl-
3-Octen-2-one
3,5-Octadien-2-one, (E,E)-
4-Acetyl-1-methylcyclohexene
3,5-Heptadien-2-one, 6-methyl-, (E)-
Acetophenone
(E)-geranyl acetone
Styrene
Phenol, 4-(2-propenyl)-
Dimethyl sulfide
Dimethyl Sulfoxide
Limonene
beta-cyclocitral
linalool
beta-ionone
beta-ionone 5,6-epoxide
dihydroactinidiolide

(B5)

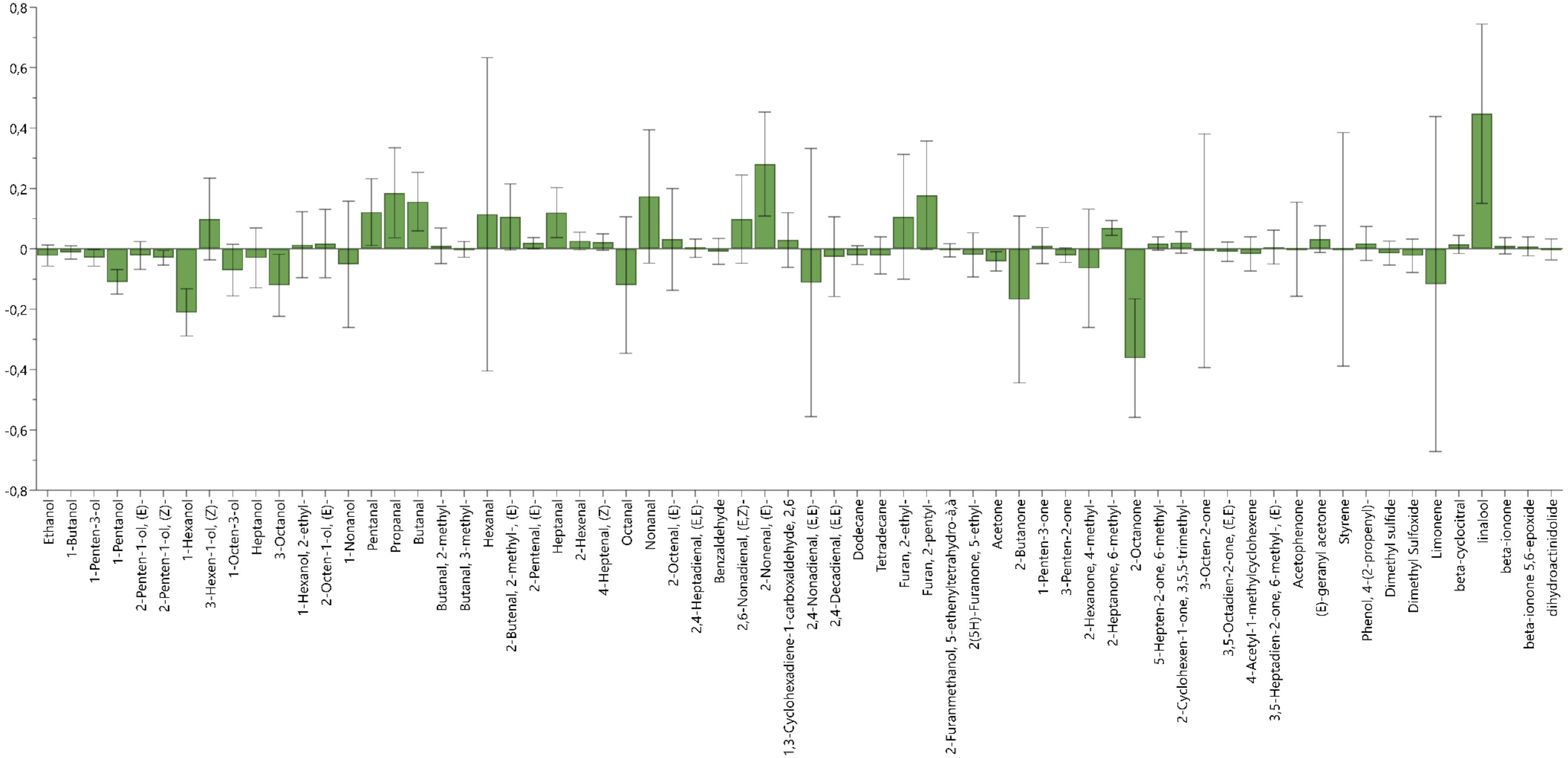
0,8
0,6
0,4
0,2
0
-0,2
-0,4
-0,6
-0,8
Ethanol
1-Butanol
1-Penten-3-ol
1-Pentanol
2-Penten-1-ol, (E)-
2-Penten-1-ol, (Z)-
1-Hexanol
3-Hexen-1-ol, (Z)-
1-Octen-3-ol
Heptanol
3-Octanol
1-Hexanol, 2-ethyl-
2-Octen-1-ol, (E)-
1-Nonanol
Pentanal
Propanal
Butanal
Butanal, 2-methyl-
Butanal, 3-methyl-
Hexanal
2-Butenal, 2-methyl-, (E)-
2-Pentenal, (E)-
Heptanal
2-Hexenal
4-Heptenal, (Z)-
Octanal
Nonanal
2-Octenal, (E)-
2,4-Heptadienal, (E,E)-
Benzaldehyde
2,6-Nonadienal, (E,Z)-
2-Nonenal, (E)-
1,3-Cyclohexadiene-1-carboxaldehyde, 2,6
2,4-Nonadienal, (E,E)-
2,4-Decadienal, (E,E)-
Dodecane
Tetradecane
Furan, 2-ethyl-
Furan, 2-pentyl-
2-Furanmethanol, 5-ethenyltetrahydro-à,à
2(5H)-Furanone, 5-ethyl-
Acetone
2-Butanone
1-Penten-3-one
3-Penten-2-one
2-Hexanone, 4-methyl-
2-Heptanone, 6-methyl-
2-Octanone
5-Hepten-2-one, 6-methyl-
2-Cyclohexen-1-one, 3,5,5-trimethyl-
3-Octen-2-one
3,5-Octadien-2-one, (E,E)-
4-Acetyl-1-methylcyclohexene
3,5-Heptadien-2-one, 6-methyl-, (E)-
Acetophenone
(E)-geranyl acetone
Styrene
Phenol, 4-(2-propenyl)-
Dimethyl sulfide
Dimethyl Sulfoxide
Limonene
beta-cyclocitral
linalool
beta-ionone
beta-ionone 5,6-epoxide
dihydroactinidolide
